# ROP-like retinopathy in full/near-term newborns: A etiology, risk factors, clinical and genetic characteristics, prognosis and management

**DOI:** 10.3389/fmed.2022.914207

**Published:** 2022-08-10

**Authors:** Limei Sun, Wenjia Yan, Li Huang, Songshan Li, Jia Liu, Yamei Lu, Manxiang Su, Zhan Li, Xiaoyan Ding

**Affiliations:** ^1^State Key Laboratory of Ophthalmology, Zhongshan Ophthalmic Center, Sun Yat-sen University, Guangzhou, China; ^2^The Sixth Affiliated Hospital of Guangzhou Medical University, Qingyuan, China; ^3^Zhuhai Maternity and Child Health Hospital, Zhuhai, China

**Keywords:** ROP-like retinopathies, full/near-term newborns, risk factor, etiology, family exudative vitreoretinopathy 3/25

## Abstract

**Purpose:**

Retinopathy of prematurity (ROP) like retinopathy (ROPLR) could occur in full/near-term newborns. The causes and clinical features are still largely elusive. This study focused on the risk factors, clinical and genetic characteristics, treatment and outcome, and prognosis of ROPLR.

**Methods:**

A total of 47 consecutive full/near-term newborns during 2016–2017 with ROPLR were included. The clinical and genetic characteristics, treatment and outcome, prognosis, and potential underlying etiology of ROPLR were were analyzed.

**Results:**

91 eyes of 47 infants were found to have ROPLR. The ROPLR regressed completely in 65.9% and partially in 20.9% of eyes without any interventions. Retinal changes of family exudative vitreoretinopathy (FEVR) were allocated in 12 neonates (group A), perinatal hypoxia-ischemia were categorized in 17 neonates (group B), and the other 18 neonates were categorized in group C. Compared to those in group B/C, infants in group A had significantly more severe retinopathy (stage 4/5, *p* < 0.001) and more treatments (*p* < 0.00 risk factor 1).

**Conclusions:**

Perinatal hypoxia-ischemia might be a major risk factor for ROPLR, in which spontaneous regression was common. FEVR, confirmed by positive family findings and genetic testing, might be the second risk factor of ROPLR, in which retinopathy is more severe and treatment is needed.

## Introduction

Retinopathy of prematurity (ROP) is a potentially severe complication of prematurity, characterized by incomplete retinal vascular development of the peripheral retina in premature infants. It is a leading cause of avoidable childhood blindness worldwide, especially in middle-income countries (1, 2). The leading factors associated with ROP are prematurity, low birth weight, and exposure to high concentrations of O_2_ (3). In full term infants, retinopathies can occur with clinical features that can appear similar to ROP (4–6). ROP-like retinopathies (ROPLR) refers to ROP developing in full/near-term neonates at a birth weight heavier than 2400 g (7). Some potential risk factors, such as brain anomalies and defects and poor maternal nutrition, increased oxygen tension in the retina after birth, hypoxic-ischemic encephalopathy (HIE) and family exudative vitreoretinopathy (FEVR) were suspected to be related to the occurrence of ROPLR (8, 9). Additionally, perinatal infection and/or inflammation had been clarified playing important etiologic roles in ROP (10). Based on current knowledge about ROP etiology and pathogenesis, it seems likely that infection and/or inflammation may be a separate insult aside of hypoxia in ROPLR.

Recently, controversy exists regarding whether ROPLR is atypical FEVR or a new clinical entity. FEVR is a congenital retinal vascular development disorder caused by disruption of the Wnt-β signaling pathway (11). The failure of peripheral retinal vascularization results in a similar appearance to the clinical spectrum of ROP. In previous studies, up to 58–76.6% of family members of patients with FEVR had abnormal fundus findings (12). Therefore, careful fundus examination of family members is important to characterize this disease. Nevertheless, to date, there has been no study focusing on fundus findings in family members of term infants with ROPLR. Therefore, the purpose of the present study was to investigate the clinical and genetic characteristics, treatment and outcome, prognosis, and potential underlying etiology of ROPLR.

## Methods

### Study cohort

This prospective study was conducted in three hospitals (including two community hospitals and a tertiary referral-based pediatric retina clinic). In the two community hospitals, universal newborn eye screening was performed in every neonate. 47 full/near-term neonates diagnosed with ROPLR from 2016–2017 were included. This study was approved by the Institutional Review Board of Zhongshan Ophthalmic Center, Sun Yat-sen University, and accorded with the tenets of the Declaration of Helsinki. Parents of all participants signed informed consent forms. Neonates who were born at 37–41 gestational weeks, with birth weight > 2000 g, were enrolled. Patients with any other congenital ocular disorders, such as persistent fetal vasculature (PFV) or incontinentia pigmenti (IP), were excluded. Handheld slit-lamp and fundus examination (RetCam III, Clarity Medical Systems, Pleasanton, CA, United States) were performed by an ophthalmologist in each neonate within 72 h after birth.

Neonatal factors included gestational age (GA), birth weight (BW), gender, race, post-natal oxygen exposure, cephalhematoma, intracranial hemorrhage, neonatal hyperbilirubinemia, umbilical cord around fetal neck (UCAN), and fetal distress. The antenatal maternal variables such as mode of delivery (vaginal or cesarean), anemia, gestational diabetes mellitus (GDM), hypertensive disorders complicating pregnancy (HDCP), turbidity of amniotic fluid, placenta previa, gestational thyroid disease (hypothyroidism or hyperthyroidism), vaginal wall laceration, perineal laceration, premature rupture of membranes, and placental abruption were recorded and reviewed.

### Assessment, treatment, and follow-up

All ophthalmic examinations were performed by qualified ophthalmologists using the 2005 International Classification of Retinopathy of Prematurity (ICROP) (13). The fundus findings of ROPLR were staged according to the international guidelines for ROP (13). The indications for treatment were based on the criteria proposed by the Early Treatment for Retinopathy of Prematurity (ETROP) Study (14). Treatments were carried out by a single surgeon (Ding X). Intravitreal injection of ranibizumab (IVR) in 0.25 mg /0.025 ml or ablative laser photocoagulation (LPC) was performed (15, 16). Surgical management is recommended for stages 4 and 5, as it might halt progression to worse complications (17). Cases with stages 1 or 2 in zone I without plus disease require a follow-up once a week, while stages 1 or 2 in zone II, zone III, or stage 3 in zone II without plus disease should be observed with 2 or 3 weekly follow-up. A repeat IVR, LPC, or surgery was considered if the clinical features were compatible with retinopathies recurrence (16). The follow-up examination was performed weekly for 1 month, biweekly for 2 months, and then less frequently in a gradual pattern, from every 4 weeks to 3 months to 6 months.

### Main outcome measures

The main outcome measures were as follows: the percentage of neonates in need of treatment and anatomical outcomes after treatment at the most recent follow-up. The outcomes were defined as the ocular structure status at the final visit according to previous studies (18); Complete regression was defined as an attached macula with full vascularization; Partial regression was defined as an absence of plus disease and neovascularization with the presence of peripheral avascular area; Stable was defined as the retinopathy with neither regression nor progression. Progression was defined as the evolution of the lesion needing treatment or with recurrence.

To investigate the potential underlying etiology and risk factors of ROPLR, a comprehensive ocular examination was performed in all the parents and siblings. All the fundus findings were graded by two experienced pediatric retinal specialists (Sun L and Yan W) to determine the presence and severity of retinopathy.

### Genetic analysis

Genomic DNA was extracted from the peripheral blood of each proband and family member using a standardized protocol. All samples underwent whole exon sequencing (WES). The method used for bioinformatics analysis of the pathogenic variants was the same as our previous study (19). Sanger sequencing was conducted to verify the mutation in the probands and family members.

### Statistical analysis

Statistical analysis was conducted using SPSS 22.0. Shapiro—Wilk tests were used to analyze the distribution of samples. Continuous variables were compared using the student's test for normally distributed variables and the Wilcoxon rank-sun test for non-normally distributed variables, and *p* < 0.05 was considered statistically 'significant.

## Results

### Demographic and clinical features of ROPLR

A total of 47 neonates and their biological parents were enrolled in this study. The cohort consisted of 35 boys and 12 girls. The male-to-female ratio was 2.9:1. The average gestational age (GA) was 38.6 ± 1.2 weeks, with a range from 37 to 41 weeks. The average birth weight was 3,087 ± 259g (2,300–3,950g). Retinopathy was found in 91 eyes of 47 infants (3 unilateral, 44 bilateral), including stage 1 lesions in 51 (54.3%) eyes, stage 2 in 20 (21.3%) eyes, stage 3 in 9 (9.5%) eyes, stage 4 in 6 (6.4%) eyes, and stage 5 in 5 (5.3%) eyes. 10 infants (21.3%) presented asymmetry with unilateral ROPLR or a difference of one stage or more between two eyes (Table 1).

**Table 1:** Demographic and clinical features, risk factors and treatment of newborns with ROP-like retinopathies.

**Case**	**Gender**	**BW(g)**	**GA(w)**	**DM**	**Risk factors**	**IVD(d)**	**Family history**	**Eye**	**Zone**	**Stage**	**Treatments**	**Follow-up(months)**	**Outcomes**	**Gene**
1	M	2,300	37	VG	None	3	P	OD	III	2	observation	36	PR	FZD4
	M	2,300	37	VG	None	3	P	OS	III	2	observation		PR	
2	M	3,100	38	VG	None	3	P	OD	NA	5	observation	54	stable	NDP
	M	3,100	38	VG	None	3	P	OS	NA	5	observation		stable	
3	M	3,100	39	VG	None	2	P	OD	NA	4b	IV scleral buckling	36	stable	FZD4
	M	3,100	39	VG	None	2	P	OS	NA	4b	IV		stable	
4	M	3,150	38	CS	None	3	P	OD	NA	4b	IV photocoagulation	60	stable	TSPAN12
	M	3,150	38	CS	None	3	P	OS	NA	4b	IV photocoagulation		stable	
5	F	3,600	38	CS	placenta previa	2	P	OD	II	2	photocoagulation	54	PR	TSPAN12
	F	3,600	38	CS	placenta previa	2	P	OS	II	2	photocoagulation		PR	
6	M	2,900	39	VG	None	2	P	OD	NA	4b	observation	50	stable	LRP5
	M	2,900	39	VG	None	2	P	OS	NA	4b	observation		stable	
7	M	3,050	38	CS	None	3	P	OD	NA	5	observation	39	stable	NDP
	M	3,050	38	CS	None	3	P	OS	NA	5	observation		stable	
8	F	3,200	40	VG	None	3	P	OD	NA	5	observation	45	stable	FZD4
	F	3,200	40	VG	None	3	P	OS	II	3	IV		PR	
9	M	2,450	38	CS	None	2	P	OD	II	2	observation	60	PR	N
	M	2,450	38	CS	None	2	P	OS	II	2	observation		PR	
10	M	3,100	40	VG	turbidity of amniotic fluid	2	P	OD	III	2	observation	60	PR	N
	M	3,100	40	VG	umbilical cord around fetal neck			OS	III	2	observation		PR	
11	M	3,300	37	CS	None	3	N	OD	III	2	observation	36	CR	N
	M	3,300	37	CS	None	3	N	OS	III	2	observation		CR	
12	F	2,500	39	VG	None	2	N	OD	III	1	observation	48	CR	N
	F	2,500	39	VG	None	2	N	OS	III	1	observation		CR	
13	M	3,000	38	VG	None	3	N	OD	III	1	observation	40	CR	N
	M	3,000	38	VG	None	3	N	OS	III	1	observation		CR	
14	M	3,200	39	VG	None	3	N	OD	III	1	observation	54	CR	N
	M	3,200	39	VG	None	3	N	OS	III	1	observation		CR	
15	F	2,500	40	CS	None	2	N	OD	III	1	observation	60	CR	N
	F	2,500	40	CS	None	2	N	OS	III	1	observation		CR	
16	M	3,300	39	VG	hyperbilirubinemia	2	N	OD	III	1	observation	60	CR	N
	M	3,300	39	VG	hyperbilirubinemia	2	N	OS	III	1	observation		CR	
17	M	3,050	40	VG	None	2	N	OD	III	1	observation	54	CR	N
	M	3,050	40	VG	None	2	N	OS	III	1	observation		CR	
18	M	3,100	39	VG	hyperbilirubinemia	1	N	OD	III	1	observation	45	CR	N
	M	3,100	39	VG	hyperbilirubinemia	1	N	OS	III	1	observation		CR	
19	F	3,350	39	CS	None	2	N	OD	III	1	observation	60	CR	N
	F	3,350	39	CS	None	2	N	OS	III	1	observation		CR	
20	M	3,330	40	VG	maternal anemia	2	N	OD	III	1	observation	60	CR	N
	M	3,330	40	VG	maternal anemia	2	N	OS	III	1	observation		CR	
21	M	2,750	37	VG	fetal distress	2	N	OD	III	1	observation	60	CR	N
	M	2,750	37	VG	fetal distress	2	N	OS	III	1	observation		CR	
22	M	2,750	37	VG	umbilical cord around fetal neck	2	N	OD	III	1	observation	60	CR	N
	M	2,750	37	VG	umbilical cord around fetal neck	2	N	OS	III	1	observation		CR	
23	M	2,950	39	VG	maternal anemia	2	N	OD	III	1	observation	52	CR	N
	M	2,950	39	VG	maternal anemia	2	N	OS	III	1	observation		CR	
24	M	3,170	38	CS	umbilical cord around fetal neck	2	N	OD	III	1	observation	60	CR	N
	M	3,170	38	CS	umbilical cord around fetal neck	2	N	OS	III	1	observation		CR	
25	M	2,650	37	CS	umbilical cord around fetal neck	1	N	OD	III	1	observation	60	CR	N
	M	2,650	37	CS	umbilical cord around fetal neck	1	N	OS	-	0	-		-	
26	F	2,750	37	CS	None	2	N	OD	III	1	observation	60	CR	N
	F	2,750	37	CS	None	2	N	OS	III	1	observation		CR	
27	M	3,200	37	CS	None	2	N	OD	III	1	observation	60	CR	N
	M	3,200	37	CS	None	2	N	OS	III	1	observation		CR	
28	M	3,300	39	CS	None	2	N	OD	III	1	observation	60	CR	N
	M	3,300	39	CS	None	2	N	OS	III	1	observation		CR	
29	M	3,300	40	VG	intracerebral hemorrhage	3	N	OD	III	1	observation	45	CR	N
	M	3,300	40	VG	intracerebral hemorrhage	3	N	OS	III	1	observation		CR	
30	M	3,550	40	VG	None	2	N	OD	III	1	observation	54	CR	N
	M	3,550	40	VG	None	2	N	OS	III	1	observation		CR	
31	M	2,700	37	VG	fetal distress	3	N	OD	II	2	IV	60	PR	N
	M	2,700	37	VG	fetal distress	3	N	OS	II	1	observation		CR	
32	M	3,200	40	CS	None	3	N	OD	III	1	observation	54	CR	N
	M	3,200	40	CS	None	3	N	OS	II	3	IV		PR	
33	M	3,500	40	CS	None	3	P	OD	II	2	observation	42	PR	N
	M	3,500	40	CS	None	3	P	OS	II	2	observation		PR	
34	F	2,980	40	CS	Intrauterine hypoxia	3	N	OD	III	3	observation	54	PR	N
	F	2,980	40	CS	Intrauterine hypoxia	3	N	OS	III	3	observation		PR	
35	M	3,000	40	VG	None	3	N	OD	III	1	observation	48	CR	N
	M	3,000	40	VG	None	3	N	OS	III	1	observation		CR	
36	M	2,900	37	CS	None	3	N	OD	III	1	observation	54	CR	N
	M	2,900	37	CS	None	3	N	OS	III	2	observation		CR	
37	M	3,500	38	CS	None	3	N	OD	III	1	observation	36	CR	N
	M	3,500	38	CS	None	3	N	OS	II	3	IV		PR	
38	F	2,500	37	CS	None	3	N	OD	III	3	observation	48	CR	N
	F	2,500	37	CS	None	3	N	OS	III	3	observation		CR	
39	F	2,530	37	CS	None	3	N	OD	III	3	observation	48	CR	N
	F	2,530	37	CS	None	3	N	OS	III	3	observation		CR	
40	M	3,700	38	VG	None	3	P	OD	II	1	observation	30	PR	N
	M	3,700	38	VG	None	3	P	OS	II	1	observation		PR	
41	M	3,450	39	VG	None	1	N	OD	II	1	observation	33	CR	N
	M	3,450	39	VG	None	1	N	OS	III	2	observation		CR	
42	F	3,950	37	VG	maternal anemia	2	N	OD	III	1	observation	42	CR	N
	F	3,950	37	VG	maternal anemia	2	N	OS	-	0	-		-	
43	M	3,500	40	VG	turbidity of amniotic fluid	1	N	OD	III	2	observation	36	CR	N
	M	3,500	40	VG	umbilical cord around fetal neck	1	N	OS	III	1	observation		CR	
44	F	2,960	41	VG	maternal anemia	2	N	OD	III	2	observation	42	CR	N
	F	2,960	41	VG	maternal anemia	2	N	OS	-	0	-		-	
45	F	3,250	40	VG	turbidity of amniotic fluid	2	N	OD	III	1	observation	33	CR	N
	F	3,250	40	VG	turbidity of amniotic fluid	2	N	OS	III	1	observation		CR	
46	M	3,330	39	VG	premature rupture of membranes	1	N	OD	II	2	photocoagulation	42	PR	
	M	3,330	39	VG	premature rupture of membranes	1	N	OS	II	2	photocoagulation		PR	N
47	M	3,170	37	VG	maternal anemia	2	N	OD	III	2	observation	36	CR	
	M	3,170	37	VG	maternal anemia	2	N	OS	III	1	observation		CR	N

F, female; M, male; BW, birth weight; GA, gestational age; DM, delivery method; VG, vaginal delivery; CS, caesarean section; RF, risk factors; IVD, initial visit date; IV, intravitreal injection; CR, complete regression; PR, partial regression.

### Treatment and outcome of ROPLR

Combined with treatment criteria and after careful discussion with parents or guardians, a total of 12 eyes of eight patients received a range of treatments, which were as follows: single IVR in five eyes of five patients, combined IVR with scleral buckling in one eye, combined IVR with LPC in two eyes of two patients, and LPC in four eyes of two patients. The other eyes were left for observation, which included in 6 eyes with stage 3 in zone III without plus disease, 2 eyes with stage 4b presented retinal folds with macula involvement and 5 eyes with stage 5 presented complete retinal detachment. All neonates were followed once a week until the retinal lesion regressed or remained stable after the treatment. The ROPLR completely regressed in 60 eyes (65.9%, 60/91) of 35 infants without any intervention, and partially regressed in 8 eyes of six infants after treatments and in 12 eyes (13.2%, 12/91) of six infants without intervention. None of the neonates presented recurrence that needed retreatment (Table 1). Eleven eyes in 6 patients with stage 4/5 severe retinopathy remained stable, including 4 eyes been treated and 7 eyes under observation. The median follow-up period was 52 months, ranging from 30 to 60 months after the first screening.

There were no statistical differences in age, gender, DM or risk factors ratio between treatment needed eyes and not requiring treatment eyes (*p* = 0.952, 1.000, 0.538, and 1.000). There were significantly larger birth weight and more gene mutations in treatment needed eyes compared to the not requiring treatment eyes (*p* = 0.035, < 0.001) (Table 2).

**Table 2:** Multivariate analysis between treatment needed eyes and not requiring treatment eyes.

	**Treatment needed eyes**	**Observation eyes**	***P* value**
Total eyes number	12	79	
Gender			1.000
Male	9 (75.0)	60 (75.9)	
Fmale	3 (25.0)	19 (24.1)	
BW(g)	3,247 ± 250.9	3,058 ± 353.9	0.035
GA(w)	38.58 ± 0.9003	38.58 ± 1.236	0.952
DM			0.538
VG	6 (50.0)	48 (60.8)	
CS	6 (50.0)	31 (39.2)	
Risk factors			1.000
Positive	5 (41.7)	30 (40.0)	
Negative	7 (58.3)	49 (60.0)	
Gene mutations			<0.001
Positive	7 (58.3)	9 (11.4)	
Negative	5 (41.7)	70 (88.6)	

BW, birth weight; GA, gestational age; DM, delivery method; VG, vaginal delivery; CS, caesarean section.

### Fundus findings in the family members in neonates with ROPLR

Typical characteristics of FEVR, including retinal peripheral avascular areas (AVA), increased ramification, and vascular straightening (Supplement Figure), were found in 18 individuals from 12 (25.5%) families. Of the 12 FEVR families, two of them had positive family history because of the affected father, the other 10 declined positive family history until the proband was diagnosed. The diagnosis of FEVR was further confirmed by vascular leakage on FFA (9) and TEMPVIA (18, temporal mid-peripheral vitreoretinal interface abnormality) on SLO. TEMPVIA, described in our prior study (20), is an image biomarker with a high sensitivity and specificity in the quick screening and diagnosis of FEVR. Briefly, it was presented as a grayish-white band located at the temporal retina, while the anterior margin was shown as the boundary of the peripheral vascular and avascular areas.

### Genetic findings in neonates with ROPLR and their family members

In total, 35 (35/47, 74.5%) neonates and their family members agreed to receive genetic analysis. Of them, 12 infants all with positive fundus findings in their family members, and the other 23 infants (23/35, 65.7%) with negative family findings. The results of WES analysis showed that 8 pathogenic mutations in 8 different pedigrees (8/47, 17.0%) were identified (Table 3). All of them were from neonates with positive family findings, revealing a positive rate of 66.7% (8 / 12).

**Table 3:** Variants of mutated genes in newborns with ROP-like retinopathies.

**Case**	**Gene**	**Genotype**	**Location**	**Cdna**	**Protein**	**Allele Type**	**Heredity**	**Reference**	**SIFT**	**Polyphen2**	**Mutation Assessor**	**PROVEAN**
1	FZD4	heterozygous	exon 2	757 C > T	R253C	Missense	paternal	Zhao et al.^20^	Damaging	Probably damaging	Medium	Damaging
3	FZD4	heterozygous	exon 1	284A>T	Q95L	Missense	paternal	Novel	Damaging	Benign	Neutral	Damaging
6	LRP5	heterozygous	exon12	2558 A >G	Gln853Arg	Missense	paternal	Novel	Damaging	Probably damaging	Medium	Neutral
7	NDP	hemizygous	exon3	362G > A	Arg121Gln	Missense	maternal	Johnson et al.^19^	Damaging	Probably damaging	Low	Damaging
2	NDP	hemizygous	exon 2	CNV		Deletion	maternal	Novel	-	-	-	-
4	TSPAN12	heterozygous	exon 4	285 + 1G > A	IVS4 dsG-A + 1	Splicing	paternal	Novel	-	-	-	-
5	TSPAN12	heterozygous	exon7	518delA	p.E173fs	Frameshift	maternal	Novel	-	-	-	-
8	FZD4	heterozygous	exon 1	c4950insCCCGGGGGCG	p.V17fs	Frameshift	maternal	Novel	-	-	-	-

Of these mutations, two different NDP mutations [deletion of exon 2,362 G > A (21)] were identified in two boys presenting as bilateral typical retinal detachment accompanied by a retrolenticular fibrotic mass. The heterozygous LRP5 mutation (2,558 A > G) was found in a two-day-old boy with stage 4 ROPLR in both eyes. The variant was found in his father, who had bilateral stage 1 ROPLR.

**Figure 1:**
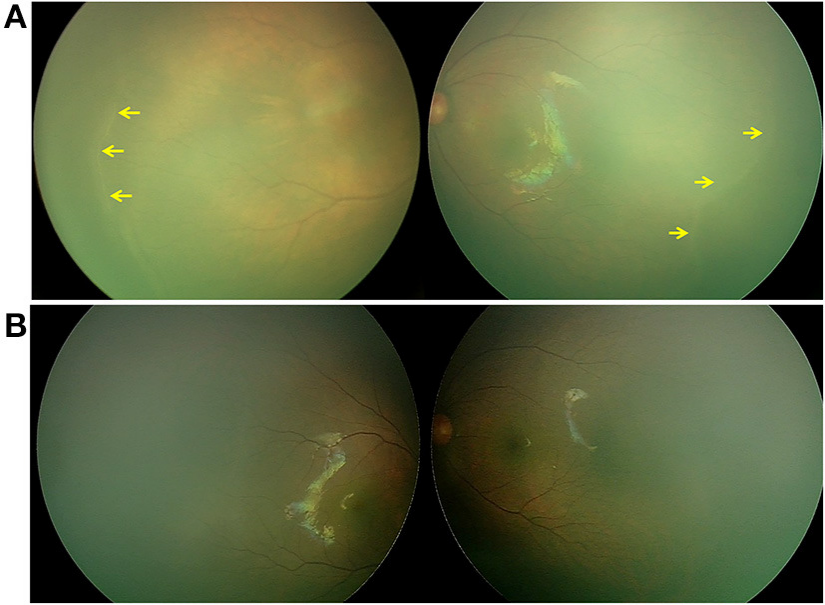
Retina imaging of a full-term newborn with FZD4 mutation (case 1, GA 37W, BW 2300g, and IVD 37W^+3^). **(A)** Fundus picture showing retinal ridges (yellow arrow) in zone II in bilateral eyes and avascular retinal periphery. **(B)** Fundus images of both eyes showing that the ridge resolved spontaneously 26 months after the first visit.

**Figure 2:**
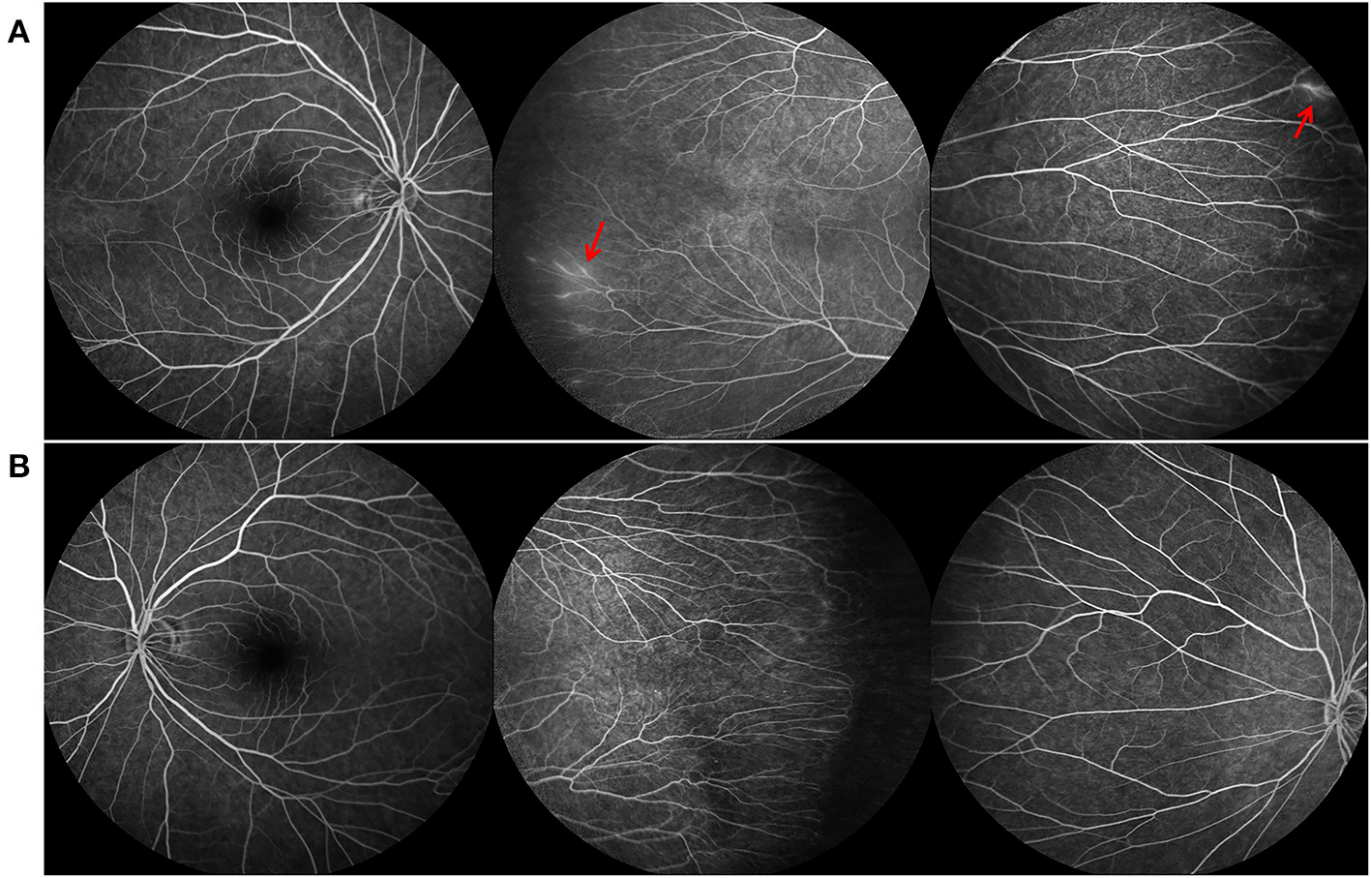
Fundus fluorescein angiography (FFA) findings of the father of case 1, who also carried the FZD4 mutation. **(A,B)** are bilateral fundus images, showing temporal avascular area with brush-shaped peripheral retinal vessles and leakage of the dye (red arrow).

**Figure 3:**
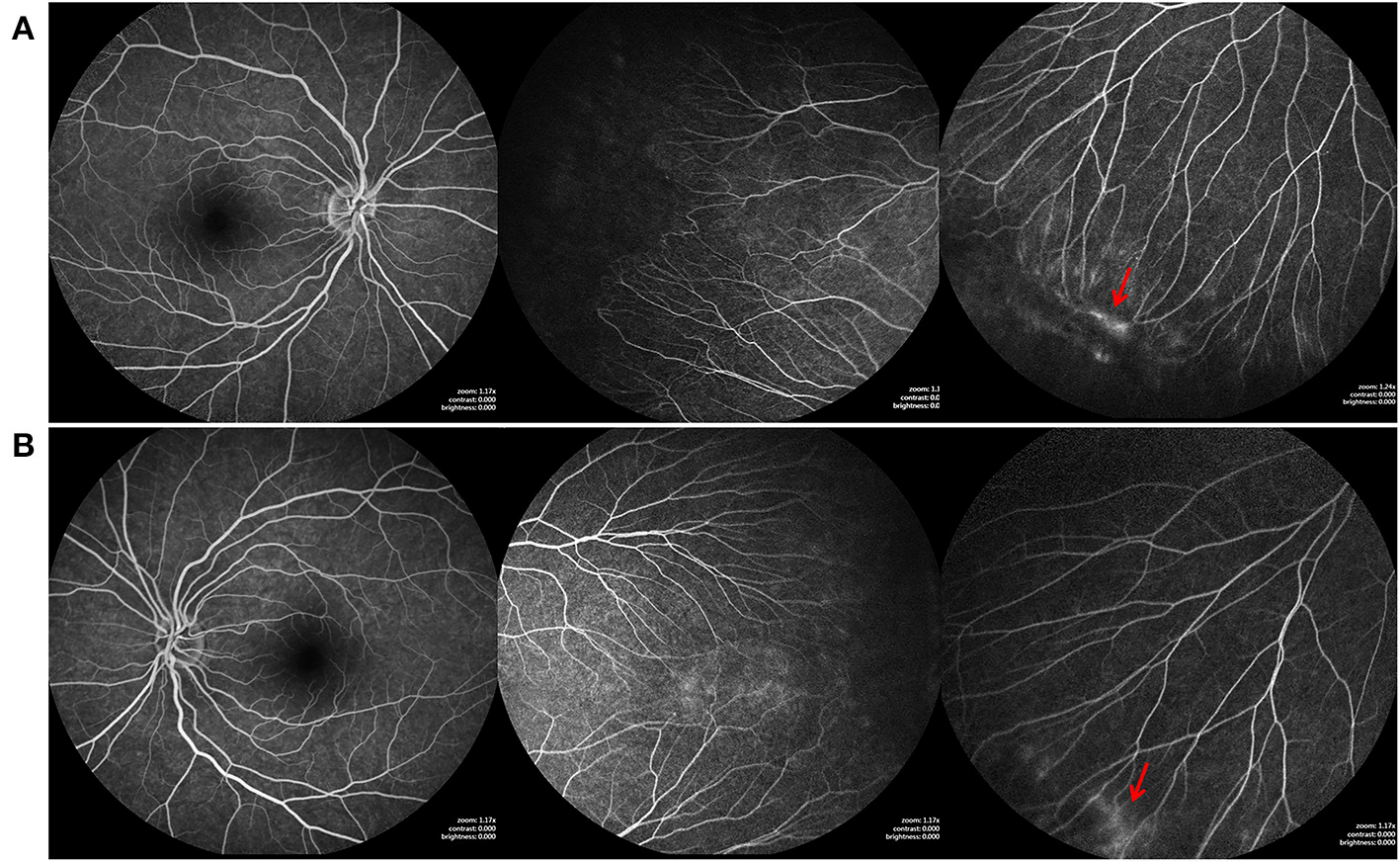
Fundus fluorescein angiography (FFA) findings of the elder sister of case1, who also carried the FZD4 mutation. **(A,B)** are bilateral fundus images, showing temporal avascular area with brush-shaped peripheral retinal vessels, avascular retinal periphery and leakage of the dye (red arrow).

There were three variants in FZD4 identified in the cohort, including two missense mutations [757C > T (22), 284A > T] and one frameshift mutation (c49–50insCCCGGGGGCG). The carrier of the variant 757C > T was a three-day-old boy who had bilateral retinal ridge and avascular retinal in the peripheral retina (Figure 1). His father was proband in his family. The variant was inherited from his father, and his sister also harbored the mutation. Although his father and sister had no clinical symptoms, peripheral avascularity of bilateral eyes were observed in FFA (Figures 2, 3). Variant c284A > T was detected in a two-day-old boy with bilateral retinal folds accompanied by vitreous hemorrhage. His father was proband in his family. The mutation was also detected in his father and sister, both of whom had peripheral avascularity and retinal folds. A two-day-old girl with c49–50insCCCGGGGGCG mutation had retinal detachment in the right eye and peripheral neovascularization in the left eye. Her mother, who carried the mutation, had stage 1 ROPLR.

Two different variants in TSPAN12, a splicing mutation (285 + 1G > A) and a frameshift mutation insertion (518delA), were included. Variant 285 + 1G > A was detected in a three-day-old boy with bilateral retinal folds. His father, who carried the same mutation, showed bilateral peripheral avascular areas. A two-day girl with 518delA mutation had bilateral retinal ridge and avascular retinal periphery. Her mother carried the mutation with bilateral peripheral avascularity. We measured no specific associations between phenotype and genotype due to the small size of the cohort.

### Perinatal hypoxia-ischemia in neonates with ROPLR

Of the 47 neonates, perinatal risk factors of 9 different variables was found in 19 infants: placenta previa in one infant, turbidity of amniotic fluid in three, umbilical cord around fetal neck in five, hyperbilirubinemia in two, maternal anemia in four, fetal distress in two, intracerebral hemorrhage in one, intrauterine hypoxia in one, and premature rupture of membranes in one. Two neonates had both turbidity of amniotic fluid and umbilical cord around the fetal neck (Table 1).

### Sub–grouping of ROPLR neonates and the analysis of the risk factors

In order to clarify the potential etiology and risk factors of ROPLR, all neonates were subdivided into three groups according to the presence of positive findings in family members and perinatal hypoxia-ischemia. Of the 47 neonates with ROPLR, 12 neonates (25.5%) whose family members presented positive fundus findings were allocated to group A, 17 neonates (three unilateral, 14 bilateral, 36.2%) with perinatal hypoxia-ischemia were categorized into group B, and the other 18 neonates (38.3%) were allocated to group C. Two neonates were both with perinatal hypoxia-ischemia, and family members presented positive fundus findings were allocated to group A. The demographic characteristics, clinical features, and treatment outcomes were compared among the groups and listed in Table 4.

**Table 4:** Sub-grouping of ROPLR neonates and the analysis of the risk factors and outcomes.

	**Group A**	**Group B**	**Group C**	**P values**
Total number, *n* (%)	12 (25.5)	17 (36.2)	18 (38.3)	**-**
Sex, *n* (%)				0.570
Male	10 (83.3)	13 (76.5)	12 (66.7)	
Female	2 (16.7)	4 (23.5)	6 (33.3)	
Birth weight, g	3,096 ± 414	3,126 ± 331	3,043 ± 359	0.800
GA at birth, weeks	38.6 ± 1.0	38.6 ± 1.4	38.5 ± 1.3	0.940
Stage at first visit (eye)	24	34	36	*P* (A-B-C) <0.001
0	0	3 (8.8)	0	*P* (A-B) <0.001
1	2 (8.3)	23 (67.6)	26 (72.2)	*P* (A-C <0.001
2	10 (41.7)	6 (17.6)	4 (11.1)	
3	1 (4.2)	2 (5.9)	6 (16.7)	
4	6 (25)	0	0	
5	5 (20.8)	0	0	
Total eyes	24	31	36	
Observation	17 (70.8)	28 (90.3)	34 (94.4)	P (A-B-C) <0.001
Treatment needed eyes	7 (29.2)	3 (9.7)	2 (5.6)	P (A-B) <0.001
LPC	2	2	0	P (A-C) <0.001
IV	2	1	2	
IV with LPC	2	0	0	
IV with SB	1	0	0	
Outcomes (eyes)	24	31	36	P (A-B-C) <0.001
CR	0	26 (80.3)	34 (94.4)	P (A-B) <0.001
PR	13 (54.2)	5 (16.1)	2 (5.6)	P (A-C) <0.001
Stable	11 (45.8)	0 (0)	0 (0)	
Progression	0(0)	0 (0)	0 (0)	
Gene mutations	8 66.7%)	0 (0)	0 (0)	
BCVA at final visit (logMAR)	1.53 ± 1.72	0.217 ± 0.109	0.167 ± 0.143	P (A-B-C) <0.001
				P (A-B) <0.001
				P (A-C) <0.001
Refractive error at final visit (D)	−2.59 ± 5.47	1.28 ± 0.604	1.42 ± 0.674	P (A-B-C) = 0.003
				P (A-B) <0.001
				P (A-C) <0.001

GA, gestational age; ROP, retinopathy of prematurity; IV, Intravitreal injection; LPC, laser photocoagulation; SB, scleral buckling CR, complete regression; PR, partial regression.

**Figure 4:**
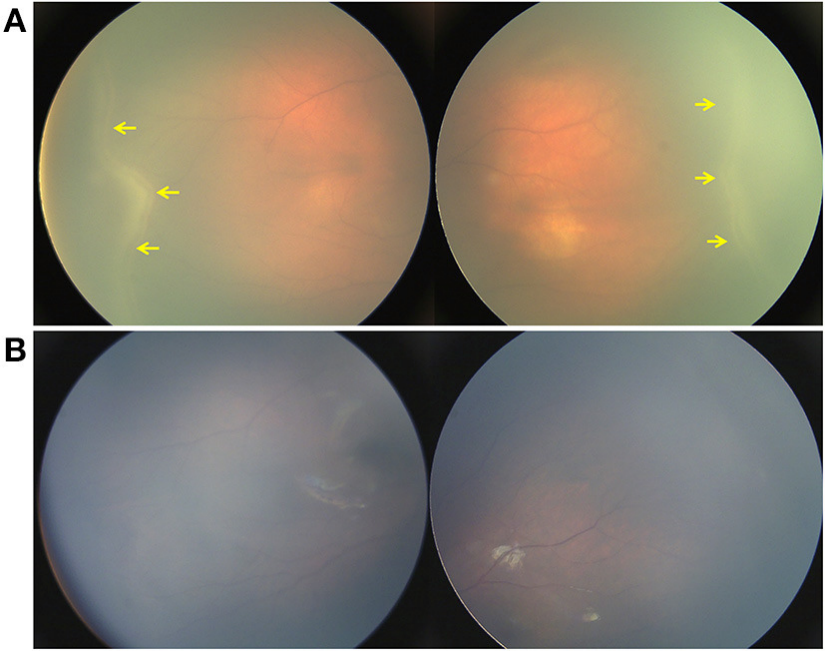
Retina imaging showing the disease course of a neonate with ROPLR (GA 37W, BW 2500g, IVD 37W^+3^). **(A)** Fundus picture showing retinal ridges and peripheral neovascularization (yellow arrow) in zone II in bilateral eyes. **(B)** Fundus images of both eyes showing that the ridges resolved spontaneously 2 months after the first visit. Neither vessel dilation nor tortuosity was found.

Essentially, there were no statistical differences in BW, age, or gender ratio among the three groups (*p* = 0.800, 0.94, and 0.57). There was no significant difference in retinopathy stage between group B and group C (*p* = 0.31). Severe retinopathy (stages 4 and 5) was seen in 45.8% (11/24) eyes in group A; however, none was found in group B and group C. Compared to those in group B and group C, infants in group A had significantly more severe retinopathy (*p* < 0.001). During the follow-up, seven eyes in group A, three eyes in group B, and two eyes in group C (29.2%, 9.7%, and 5.6%) received treatments, respectively. There were significantly more eyes in need of treatment in group A compared to group B and C (*p* < 0.001). Spontaneous complete regression was observed in 26 eyes of 15 neonates (83.9%, 26/31) in group B and in 34 eyes of 18 neonates (94.4%, 34/36) in group C (Figure 4); however, there was no one in group A (*p* < 0.001). For those 12 eyes needing treatment, eight eyes with moderate retinopathy (stage 2/3) resulted in partial regression, while 4 eyes staged 4b stayed stable. The average BCVA (logMAR) at final follow up were 1.53 ± 1.72, 0.217 ± 0.109, 0.167 ± 0.143 in group A, group B, and group C. There was significantly worse vision in group A compared to group B and C (*p* < 0.001). The refractive error at final visit were −2.59 ± 5.47D, 1.28 ± 0.604D, and 1.42 ± 0.674D in group A, group B, and group C. The prevalence of myopia was significantly higher than that in group B and group C (*p* < 0.001).

## Discussion

Retinal vascular changes similar to ROP in healthy full/near-term infants with a normal birth weight were sporadically reported in previous studies (5, 7). And among them, most of infants were with supplementary oxygen exposure. The clinical findings were classically described as “ROP-like retinopathy (ROPLR)” with an incidence between 0.42 and 0.9% of healthy babies (4, 23). It has been reported that the pathogenesis of ROPLR is associated with perinatal hypoxia-ischemia (8, 24). A study revealed that meconium-stained amniotic fluid was identified as determinants of birth asphyxia (25). However, there is no consensus regarding the etiology or diagnostic criteria for ROPLR. We herein reported 47 neonates with ROPLR who were recruited from their birth and followed up for at least 30 months. The clinical features, natural history, treatment and outcome, prognosis, genetic testing, and perinatal hypoxia-ischemia were reviewed and analyzed.

Our study is the first report providing clinical findings, treatment outcomes, and prognosis in neonates with ROPLR. Of the total 47 neonates, the clinical features varied from stage 1 to 5 according to ROP staging criteria. 10 infants presented asymmetry. The ROPLR spontaneously regressed in 79.1% infants without any intervention. According to the ROP criteria, 12 eyes of eight patients received treatments in our study and 8 eyes partially regressed and 4 eyes stable. It was encouraging that the prognosis of ROPLR was favorable after prompt treatment. No recurrence or retreatment was presented during a long follow-up period.

According to our previous studies, TEMPVIA revealed by SLO, was found in 88.3% of FEVR patients (20). Our current study has proved further that TEMPVIA, identified in 25.5% of families, could serve as a biomarker. FEVR is a rare disorder of retinal blood vessel development, leading to incomplete vascularization of the peripheral retina and poor vascular differentiation. Most patients have an avascular peripheral retina but expressivity may be asymmetric and is highly variable, ranging from asymptomatic to severe within the same family. Several genes, including FZD4, LPR5, TSPAN12, and NDP, are important components in the Wnt/Norrin signaling pathway that regulates developmental retinal angiogenesis and maintains retinal blood vessel integrity (26–29). Overall, about 44% of FEVR patients could be explained by these four gene mutations. It is noteworthy that 66.7% of these 12 families in our current cohort were confirmed carrying mutated FEVR-related gene mutations, which indicated that at least 17.0% (8/47) neonates with ROPLR actually have FEVR, and this can be confirmed clinically and genetically. Though studies have mentioned that ROP can be developed in near-term infants with normal BW (30) and neonates could behave like FEVR patients but were also with ROP, termed ROPER (ROP vs. FEVR) (31, 32), carefully evaluation of the family members and genetic analysis are essential for making the final diagnosis. Therefore, we suggest that fundus examination in family members is necessary, sensitive, and helpful for distinguishing FEVR from ROPLR. In general, family history and deliberate fundus examination, not only in babies, but also in their family members, are pivotal for identifying the underlying reasons for ROPLR.

Differential diagnosis of ROPLR is difficult but of great importance. In this study, 12 neonates were diagnosed as FEVR, in which 8 neonates had mutations and 4 neonates had no mutations. Among the 8 neonates with mutations, 6 (75%) had retinal detachment, and 5 had retinal detachment bilaterally. However, the 4 neonates without mutations had stage 1 or 2 FEVR. This result filled in the blanks of the current understanding of FEVR in neonates. However, it is impossible to accurately assess the progression of FEVR to a late stage. Further analysis of novel causative genes may explain the mild clinical findings. Our results showed that retinal folds as well as total retinal detachment could occur for several days after birth (1–3 days). FEVR in young children tends to be very aggressive, usually leading to severe complications (33). No eyes with FEVR could regress without any intervention, while seven eyes progressed to needing treatment in this study. Fortunately, with timely treatments, including IVR, LPC, and scleral buckling, satisfactory outcomes were achieved in most neonates. Severe complications, including total retinal detachment, cataracts, keratopathy, and glaucoma, were not noted in any of the patients receiving timely treatment. However, the development of visual status and refractive error were significantly affected. Therefore, early detection and prompt therapy can restore vision in a timely manner as well as prevent blindness for neonates with FEVR.

The diagnosis of real “ROPLR” can only be made after exclusion of other retinopathies in infants, including PFV, IP, and FEVR. Although the pathogenesis of ROPLR remains elusive, systemic abnormalities such as brain anomalies and defects and poor maternal nutrition, increased oxygen tension and metabolic demands in the retina after birth, and blockage of placental transfer of nutritional factors have been hypothesized as potential reasons for real ROPLR (8, 34, 35). Nineteen neonates (40.4%) with perinatal hypoxia-ischemia were discovered in the study. Twenty-eight of the 31 eyes (90.3%) spontaneously regressed in group B as the neonates grew up and the physiological situation improved. Therefore, we supposed that ROPLR might be associated with perinatal hypoxia-ischemia.

The limitations of our study lie in the limited number of enrolled patients. Moreover, the constitution of FEVR (25.5%) and ROPLR (74.5%) in term neonates might not be representative of the universal population. It should also be noted that perinatal infection/inflammatory and the interactions among risk factors need to be further explored. Therefore, further studies with larger sample sizes are necessary to validate our findings.

In conclusion, we observed 47 neonates with ROPLR. The clinical features mimicking ROP varied widely from stage 1 to stage 5. With timely screening and treatment in these neonates, the prognosis of ROPLR was favorable and encouraging, and childhood blindness might be prevented. Perinatal maternal and/or neonatal hypoxic-ischemia might be the major risk factors of ROPLR, in which the retinopathy spontaneously regressed as the physiological situation improved. FEVR, which is confirmed by positive family findings and genetic testing, might be one of the aetiologic factors of ROPLR. The retinopathy due to FEVR was more severe, and treatment was needed. It is suggested that all neonates should receive universal eye screening within 72 h of birth. Retinal detachment in a newborn suggests another mechanism other than “ROP”. Genetic testing and carefully evaluation of the family members should be prompted when a full/near-term newborn presented with retinal fold or retinal detachment. Importantly, with timely recognition and treatment in these neonates, childhood blindness may be prevented. Furthermore, studies with a larger sample size are required to confirm our findings and explore the underlying mechanism of ROPLR.

## Data availability statement

The original contributions presented in the study are included in the article/Supplementary material, further inquiries can be directed to the corresponding author.

## Ethics statement

The studies involving human participants were reviewed and approved by Institutional Review Board of Zhongshan Ophthalmic Center, Sun Yat-sen University. Written informed consent to participate in this study was provided by the participants' legal guardian/next of kin. Written informed consent was obtained from the individual(s), and minor(s)' legal guardian/next of kin, for the publication of any potentially identifiable images or data included in this article.

## Author contributions

LS, WY, and XD conceptualized and designed the study, drafted the initial manuscript, and reviewed and revised the manuscript. LH, SL, MS, JL, YL, and ZL designed the data collection instruments, collected data, carried out the initial analyses, and reviewed and revised the manuscript. All authors contributed to the article and approved the submitted version.

## Funding

This study is supported in part by grants from the Fundamental Research Funds of State Key Laboratory of Ophthalmology, Research Funds of Sun Yat-sen University (15ykjxc22d; Guangzhou, Guangdong, China), Science and Technology Program Guangzhou, China (201803010031; Guangzhou, Guangdong, China).

## Conflict of interest

The authors declare that the research was conducted in the absence of any commercial or financial relationships that could be construed as a potential conflict of interest.

## Publisher's note

All claims expressed in this article are solely those of the authors and do not necessarily represent those of their affiliated organizations, or those of the publisher, the editors and the reviewers. Any product that may be evaluated in this article, or claim that may be made by its manufacturer, is not guaranteed or endorsed by the publisher.

## Supplementary material

Supplement Figure Representative images of FEVR (case 4, GA 38W, BW 3100g, IVD 38W^+3^).
