# Supplementary figures and images for: ROP-like retinopathy in full/near-term newborns: A etiology, risk factors, clinical and genetic characteristics, prognosis and management
Source: Front Med (Lausanne). 2022 Aug 10;9:914207. doi: 10.3389/fmed.2022.914207 (PMC9399493; doi:10.3389/fmed.2022.914207)

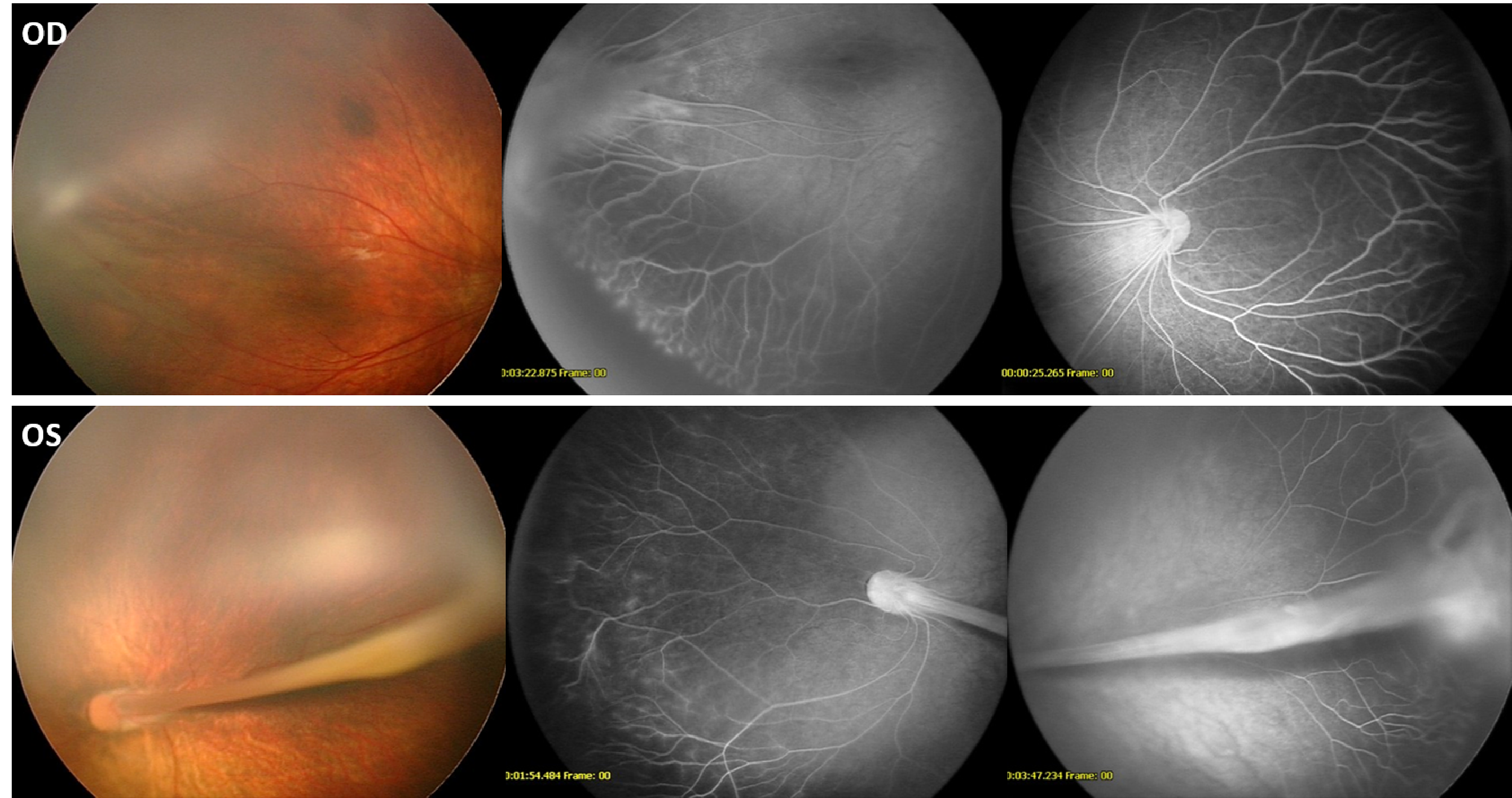

Supplement: Supplement Figure — Representative images of FEVR (case 4, GA 38W, BW 3100g, IVD 38W+3). [file Image_1.TIF]
